# Empathy among undergraduate medical students: A multi-centre cross-sectional comparison of students beginning and approaching the end of their course

**DOI:** 10.1186/s12909-016-0603-7

**Published:** 2016-03-15

**Authors:** Thelma A Quince, Paul Kinnersley, Jonathan Hales, Ana da Silva, Helen Moriarty, Pia Thiemann, Sarah Hyde, James Brimicombe, Diana Wood, Matthew Barclay, John Benson

**Affiliations:** Primary Care Unit, Department of Public Health, Primary Care University of Cambridge, IPH, Robinson Way, Cambridge, CB2 0SR UK; Institute of Medical Education, Medical School, Cardiff University 2nd floor, Neuadd Meirionydd, Heath Park, Cardiff, CF14 4XN UK; Department of Medical and Social Care Education, University of Leicester, University Road, Leicester, LE1 7RH UK; College of Medicine, University of Swansea, Swansea, Wales SA2 8PP UK; Department of Primary Health Care and General Practice, University of Otago, Wellington, 23a Mein Street, PO Box 7343, Wellington, New Zealand; School of Clinical Medicine, University of Cambridge, Box 111 Cambridge Biomedical Campus, Cambridge, CB2 0SP UK

**Keywords:** Medical students, Undergraduate, Empathy, UK, New Zealand, Jefferson Scale of Empathy-Student Version, Interpersonal Reactivity Index

## Abstract

**Background:**

Although a core element in patient care the trajectory of empathy during undergraduate medical education remains unclear. Empathy is generally regarded as comprising an affective capacity: the ability to be sensitive to and concerned for, another and a cognitive capacity: the ability to understand and appreciate the other person’s perspective. The authors investigated whether final year undergraduate students recorded lower levels of empathy than their first year counterparts, and whether male and female students differed in this respect.

**Methods:**

Between September 2013 and June 2014 an online questionnaire survey was administered to 15 UK, and 2 international medical schools. Participating schools provided both 5–6 year standard courses and 4 year accelerated graduate entry courses. The survey incorporated the Jefferson Scale of Empathy-Student Version (JSE-S) and Davis’s Interpersonal Reactivity Index (IRI), both widely used to measure medical student empathy. Participation was voluntary. Chi squared tests were used to test for differences in biographical characteristics of student groups. Multiple linear regression analyses, in which predictor variables were year of course (first/final); sex; type of course and broad socio-economic group were used to compare empathy scores.

**Results:**

Five medical schools (4 in the UK, 1 in New Zealand) achieved average response rates of 55 % (*n* = 652) among students starting their course and 48 % (*n* = 487) among final year students. These schools formed the High Response Rate Group. The remaining 12 medical schools recorded lower response rates of 24.0 % and 15.2 % among first and final year students respectively. These schools formed the Lower Response Rate Group. For both male and female students in both groups of schools no significant differences in any empathy scores were found between students starting and approaching the end of their course. Gender was found to significantly predict empathy scores, with females scoring higher than males.

**Conclusions:**

Participant male and female medical students approaching the end of their undergraduate education, did not record lower levels of empathy, compared to those at the beginning of their course. Questions remain concerning the trajectory of empathy after qualification and how best to support it through the pressures of starting out in medical practice.

## Background

The Francis Report on a failing hospital in the United Kingdom highlighted the importance of empathy to patient care [1]. Doctors’ empathy may influence clinical outcomes by facilitating trust and openness, more accurate diagnosis, shared decision making and adherence to medical recommendations [2–4]. Patients report higher levels of satisfaction, comfort and self-efficacy when they perceive a doctor to be more empathetic [5–8]. Studies have also found beneficial associations between physician empathy and physiological outcomes [9–12].

Empathy has been described as a multi-dimensional construct, comprising two main domains: an affective capacity to be sensitive to and concerned for another person; and a cognitive capacity to understand and appreciate the other person’s perspective [13, 14]. In a clinical context, it has been suggested that the cognitive component also includes the ability to communicate that understanding [15]. Empathy is considered to be normally distributed amongst the general population, whilst studies both of the general population and of medical students suggest that females are more empathetic than males [16, 17].

A widely expressed view is that medical student empathy declines during undergraduate education and that there is a need for initiatives to prevent this [18–23]. However, recently this view has been subject to debate, with some studies reporting a decline, but others showing no change [24–34]. Studies of other student health care professionals have produced similarly conflicting results [35–38].

Difficulties in measurement may be partly responsible for this mixed picture. Systematic reviews of research on empathy in medicine have highlighted problems relating to the variety and number of measures used, the failure to present evidence supporting their reliability and validity and the failure to indicate whether the affective or cognitive aspects of empathy are being addressed [39, 40]. Most studies of medical students involve self-report questionnaires which reflect personal perceptions rather than actual behaviour in clinical encounters, the latter being recorded only rarely [17, 41]. Although self-reported measures of empathy have potential biases, the psychometric properties of two, the Jefferson Scale of Empathy–student version (JSE-S) and Davis’s Interpersonal Reactivity Index (IRI), have been found to be very robust [16, 39, 40, 42].

The Jefferson Scale of Empathy–student version (JSE-S) has been widely used in many countries to measure medical student empathy [18, 28, 29, 31, 32, 43]. It was specifically developed for use in healthcare and focuses strongly on the doctor’s or healthcare practitioner’s relationship with the patient. Its originator considered clinical empathy, as measured by the JSE-S to be primarily cognitive [44].

Davis’s Interpersonal Reactivity Index (IRI) has been used with the general population and among US college students [13, 45]. It has also been used to examine clinical conditions affecting social functioning and emotions [46, 47], the development of prosocial behaviour [48, 49], the neurological basis of cognitive and affective empathy [50] and the assessment of juvenile and sex offenders [51]. The IRI is seen to measure generic empathy and although used less frequently to measure medical student empathy, it allows for comparison with other groups. The IRI comprises four subscales, two of which measure empathy directed towards others. Perspective Taking (IRI-PT) assesses consideration for the psychological point of view of the other person (cognitive empathy), whilst Empathetic Concern (IRI-EC) assesses consideration for their feelings and concerns (affective empathy).

Previously we undertook a longitudinal study, examining four cohorts of undergraduate medical students, on an annual basis, as they progressed through their course. We examined how the same students’ scores changed over time and compared scores of students in different cohorts. We found no change in medical student empathy either longitudinally or cross-sectionally [26]. However the study was conducted in a single institution and only used the IRI, hence questions remained about the findings’ generalisability.

We decided therefore to contextualise the findings of our study by undertaking a cross-sectional comparison of students at the beginning and end of undergraduate medical training, in multiple medical schools. We used the IRI (measuring cognitive and affective empathy) and the JSE-S (measuring predominantly cognitive empathy). Our research questions were:

1. Do medical students approaching the end of their undergraduate education record lower levels of empathy than medical students at the beginning of their course?
2. Do male and female students differ in this respect?
3. Do students on different entry schemes differ in this respect?

## Methods

We adopted a cross-sectional design. All medical schools in the UK were approached. Two non-UK schools, one in New Zealand and one in Ireland, expressed an interest in participating. Schools provided a range of course styles regarding primary focus on biomedical sciences in early years, integration of biomedical science with clinical experience and educational delivery, for example problem-based learning. Given the variability of these characteristics across schools and different years, it was not possible to stratify schools meaningfully in this regard. Participating schools provided both 5–6 year standard courses and 4 year accelerated graduate entry courses. Graduate course entry students could be considered comparable to US counterparts, in that they have previously completed at least a first degree (BA or BSc) and hence are normally aged 21 or over at course entry.

Participating medical schools invited all first and final year students to complete an online questionnaire. (For the New Zealand medical school, some students enter undergraduate doctor training at the end of a year-long health care foundation course, hence some invited students were entering their second year.) All schools invited final year students at some point in that academic year, depending upon local considerations.

The questionnaire measured empathy using both the JSE-S and the IRI and recorded biographical details (Table 1). Other questionnaire items, not reported here, measured psychological well-being, death anxiety and attitudes towards end of life care. Cognitive empathy was measured by the ‘Perspective taking’ IRI (IRI-PT) subscale and by the total JSE-S score. Affective empathy was measured by the ‘Empathetic concern’ IRI (IRI-EC) subscale. Participation was voluntary with no inducements offered.

**Table 1:** Empathy Measures used in the Study

Instrument	Subscales	No of items	Scored	Basis of scoring
Jefferson Scale of Empathy	JSE-S (Student version)	20	1-7 (max score 140)	Agreement with statements
Interpersonal Reactivity index	IRI-EC Empathetic Concern (Affective)	7	0-4 (max score 28)	How well each statement describes self
	IRI-PT Perspective Taking (Cognitive)	7	0-4 (max score 28)	How well each statement describes self

First year students (some second year students in New Zealand) received the survey at the beginning of the first term of the 2013 academic year (2014 in New Zealand). The survey was available to them for a mean duration of 45 days. Final year students received the survey during the first or second term of the 2013 academic year (2014 in New Zealand). The survey was available to them for a mean duration of 49 days. All schools sent out at least one reminder. The number and nature of reminders varied from school to school depending on the terms of the ethical approval granted in each institution.

Students could only access the online questionnaire by using a unique “token” or PIN. The research team in Cambridge provided randomly generated tokens to each participating school, for distribution to students. Schools randomly allocated these tokens to individual students, inviting them to participate. Completed questionnaires could only be accessed by the Cambridge team who replaced student tokens with unique, randomly generated, study identifiers. This procedure ensured that the names of students who responded were unknown both to participating schools and to the research team in Cambridge.

The Cambridge team converted raw data into scales and analysed national data using IBM SPSS version 21, Chicago. Data from their respective students were returned to participating schools. Participating schools commented on the extent to which students participating in the survey were representative of their year group. Ethical approval for the overall study was obtained from the University of Cambridge Psychology Research Ethics Committee (reference number 2012.44) and from the relevant committee in each participating medical school. (Details of ethical approval obtained from participating medical schools are presented in appendix 1.) Permission to use the JSE-S was held by the University of Cambridge and for this particular study was confirmed retrospectively.

We compared first and final year students using chi squared tests for categorical variables (biographical characteristics). We analysed the IRI subscales (IRI-PT and IRI-EC) separately and analysed the total score for the JSE-S [13, 44]. We used multiple linear regression analysis to examine the extent to which empathy scores recorded by students approaching the end of their undergraduate course were different from those recorded by students starting their course. The predictor variables in our models included: year of course (first/final); sex; type of course (UK accelerated graduate entry/UK standard entry/non-UK); and broad socio-economic group (HMO class 1/lower than class 1). Statistical significance was set at *p* = 0.05. We used Cohen’s d to estimate the effect size and therefore practical significance of statistically significant differences, adopting the convention where values of 0.2, 0.5 and 0.8 indicate small, medium and large effect sizes respectively [45].

In total, 15 UK schools and 2 non-UK schools participated. Response rates varied, ranging from 7 % to 77 % across schools and within schools between years. We adopted a pragmatic cut off of 30 % response rate in both first and final years to include schools for primary data analysis. Four UK schools and 1 school in New Zealand achieved response rates above this cut-off. These schools were termed the High Response Rate Group (HRRG). Our primary analysis focused on the results from these HRRG schools. The remaining 12 schools were termed the “Lower Response Rate Group” (LRRG). Comparisons between respondents in the HRRG and LRRG schools are presented in appendix 2.

## Results

### High response rate group schools (HRRG)

#### Response rates and demographic characteristics of respondents

Among the 4 UK HRRG schools, 3 offered both standard (5/6 year) and accelerated graduate entry (4 year) courses and 1 offered only an accelerated graduate entry course. The New Zealand school did not offer an accelerated graduate entry course.

Across the 5 HRRG schools, of 1188 possible respondents to the first/second year survey, 652 (54.9 %) responded; and of 1012 possible respondents to the final year survey, 487 (48.1 %) responded. Response rates in individual schools varied from 36 % to 77 % among first/second year students and from 31 % to 68 % among final year students (Table 2).

**Table 2:** Demographic characteristics of HRRG schools’ first/second and final year respondents

	First/second	Final
	Year	Year
All possible respondents	1188	1012
Female students	643 (54.1 %)	566 (55.9 %)
Graduate Entry students	152 (12.8 %)	148 (14.6 %)
All respondents	652	487
Female students	399 (61.2 %)	296 (60.7 %)
Graduate entry students	101 (15.5 %)	82 (16.8 %)
New Zealand students	165 (25.3 %)	140 (28.7 %)
Average Age (Years)		
Standard entry UK	18.7	25.1
Graduate entry UK	23.8	28.1
New Zealand	20.5	23.4
Socio-economic group		
HMO 1.1 & 1.2^1^	521 (80.2 %)	373 (76.6 %)

^1^UK National Statistics Socio-economic Classification: HMO: Higher Managerial Occupations eg company directors, doctors, teachers

In both first/second and final years, female and graduate entry students were slightly over-represented among respondents compared to their year group. Similar comparisons in respect of socio-economic group were not possible (Table 2).

Amongst respondents, no significant differences were found between first/second and final year in respect of gender or course type, or socioeconomic class. (Table 2).

#### Empathy scores recorded by first/second and final year students

The mean scores for the JSE-S, IRI-PT and IRI-EC recorded by first/second and final year students showed no significant difference (Table 3).

**Table 3:** Mean scores for empathy recorded by HRRG schools’ first/second and final year students by gender

	All		Female Students		Male Students	
	First/second	Final	First/second	Final	First/second	Final
	*n* = 652	*n* = 487	*n* = 399	*n* = 296	*n* = 253	*n* = 191
Instrument	Mean score (SD)	Mean score (SD)	Mean score (SD)	Mean score (SD)	Mean score (SD)	Mean score (SD)
IRI-EC	21.00 (3.961)	21.06 (4.393)	21.70 (3.643)	22.05 (4.025)	19.89 (4.188)	19.53 (4.507)
IRI-PT	19.06 (4.373)	19.31 (4.362)	19.43 (4.208)	19.14 (4.338)	18.47 (4.569)	19.58 (4.398)
JSE-S	113.03 (10.295)	113.03 (11.468)	114.94 (9.917)	114.58 (10.931)	110.02 (10.179)	111.36 (12.021)

At each time point, female students recorded significantly higher scores for JSE-S and IRI-EC than male students. However there was no significant difference in scores for any measure of empathy, between students in the first/second year and final year, amongst either males or females.

Similarly, at each time point, graduate entry course students recorded significantly higher scores for all measures of empathy than standard entry course students. But again, no differences in scores for any measure of empathy were recorded between students in the first/second and final year, amongst either graduate or standard entry course students.

### Multiple linear regression

The results of the multiple linear regression analysis confirmed these findings. Gender and type of course were confirmed as significant predictors of level of empathy. However, no significant differences in scores were found for any measure of empathy between first/second year and final year students (Table 4).

**Table 4:** Results of multiple linear regression analysis, HRRG schools’ students

Mean score difference	IRI-EC			IRI-PT			JSE-S		
	Estimate	95 % CI	*P*	Estimate	95 % CI	*P*	Estimate	95 % CI	*P*
Year of course			0.856			0.363			0.833
Year 1	−0.04	(−0.52, 0.43)		−0.24	(−0.75, 0.27)		−0.13	(−1.36, 1.09)	
Final year	Ref			Ref			Ref		
Sex			<0.001			0.174			<0.001
Female	Ref			Ref			Ref		
Male	−2.13	(−2.60, −1.65)		−0.36	(−0.88, 0.16)		−4.17	(−4.17, −2.93)	
Course type			0.003			0.007			<0.001
UK - standard entry	Ref			Ref			Ref		
UK - graduate entry	0.97	(0.32, 1.63)		1.14	(0.43, 1.86)		3.26	(1.55, 4.97)	
Non UK Medical School	0.09	(−0.46, 0.64)		0.11	(−0.49, 0.70)		4.56	(3.14, 5.98)	
Socioeconomic group			0.951			0.446			0.705
HMO 1.1 & 1.2	Ref			Ref			Ref		
Other NS-SEC groups	0.02	(−0.55, 0.59)		−0.24	(−0.86, 0.38)		−0.29	(−0.29, 1.20)	

Null hypothesis: no difference in scores by factor

### Lower response rate schools (LRRG)

#### Response rates and demographic characteristics of respondents

Among the 12 LRRG schools, 5 UK schools offered both standard (5/6 year) and accelerated, graduate entry (4 year) courses.

Across the 12 LRRG schools, of the 3009 possible respondents to the first year survey 721 (24.0 %) responded and of the 3065 possible respondents to the final year survey 476 (15.2 %) responded. Response rates in individual schools varied from 7 % to 45 % among first year students and from 7 % to 35 % among final year students. (Table 5).

**Table 5:** Demographic characteristics of LRRG schools’ first/second and final year respondents

	First/second Year	Final Year
All possible respondents	3009	3065
Female students	1704 (56.6 %)	1753 (57.2 %)
All respondents	721 (24.0 %)	476 (15.5 %)
Female students	439 (60.9 %)	298 (63.8 %)
Graduate entry students	58 (8.0 %)	26 (5.6 %)
Non UK medical school students	76 (10.5 %)	34 (7.3 %)
Average Age (Years)		
Standard entry UK	19.1	24.1
Graduate entry UK	26.1	27.7
Non UK medical school students	19.2	24.7
Socio-economic group		
HMO 1.1 & 1.2^a^	570 (79.2 %)	377 (80.7 %)

^a^UK National Statistics Socio-economic Classification: HMO: Higher Managerial Occupations eg company directors, doctors, teachers

Compared with their year group female students, were over-represented among respondents, particularly among final year students (Table 5). It was not possible to make similar comparisons in respect of graduate entry students.

Amongst LRRG respondents, small differences were found between first/second and final year in respect of gender and course type, but none in respect of socioeconomic class (Table 5).

#### Empathy scores recorded by first/second and final year students

**Table 6:** Mean scores for empathy recorded by LRRG schools’ first and final year students by gender

	All		Female Students		Male Students	
	First	Final	First	Final	First	Final
	*n* = 721	*n* = 467	*n* = 439	*n* = 298	*n* = 282	*n* = 169
Instrument	Mean score (SD)	Mean score (SD)	Mean score (SD)	Mean score (SD)	Mean score (SD)	Mean score (SD)
IRI-EC	21.25 (3.946)	21.22 4.067	21.67 (3.809)	21.96 (3.639)	20.60 (4.071)	19.92 (4.447)
IRI-PT	19.45 (4.201)	19.58 4.401	19.60 (4.008)	19.85 (4.311)	19.23 (4.483)	19.11 (4.531)
JSE-S	112.94 (10.583)	112.55 (11.922)	114.10 (9.983)	114.11 (10.852)	111.13 (11.237)	109.79 (13.194)

Among LRRG students no significant difference in scores for any measure of empathy were found between students in the first/second year and final year, amongst either males or females (Table 6).

#### Multiple linear regression

The results of the multiple linear regression analysis confirmed no difference in empathy scores recorded by students completing and starting their course. Gender was a significant predictor of all empathy scores with males scoring lower than females. However, course type did not figure as a factor influencing empathy scores. Socioeconomic group had a weak influence in respect of IRI-PT only (Table 7).

**Table 7:** Results of multiple linear regression analysis, LRRG schools’ students

Mean score difference	IRI-EC			IRI-PT			JSE-S		
	Estimate	95 % CI	*P*	Estimate	95 % CI	*P*	Estimate	95 % CI	*P*
Year of course			0.776			0.621			0.528
Year 1	0.66	(−0.39, 0.53)		−0.13	(−0.62, 0.37)		0.41	(−0.87, 1.70)	
Final year	Ref			Ref			Ref		
Sex			<0.001			0.027			<0.001
Female	Ref			Ref					
Male	−1.48	(−1.94, −1.02)		−0.57	(−1.07, −0.07)		−3.51	(−4.80, −2.22)	
Course type			0.496			0.268			0.957
UK - standard entry	Ref			Ref			Ref		
UK - graduate entry	0.53	(−0.35, 1.41)		0.79	(−0.17, 1.74)		0.37	(−2.08, 2.83)	
Non UK Medical School	−0.14	(−0.91, 0.64)		−0.33	(−1.17, 0.51)		0.32	(−1.85, 2.48)	
Socioeconomic group			0.100			0.049			0.608
HMO 1.1 & 1.2	Ref			Ref			Ref		
Other NS-SEC groups	0.47	(−0.09, 1.03)		0.61	(0.00, 1.21)		0.41	(−1.15, 1.97)	

Null hypothesis: no difference in scores by factor

### Comparisons between students in the HRRG and LRRG schools

There were proportionately fewer graduate entry students among respondents in the LRRG schools as compared to respondents in the HRRG schools. No significant differences were found in respect of gender, age, or socio-economic group (Appendix 2, Table 8).

First year male students in the LRRG schools recorded slightly higher scores for IRI-EC than their HRRG counterparts. Similarly final year female students in the LRRG schools recorded slightly higher scores for IRI-PT (Appendix 2).

## Discussion

Medical students approaching the end of their course in the four UK medical schools and one New Zealand school, irrespective of gender or course type, showed similar results for both affective and cognitive empathy to students beginning their course. These results were confirmed by multiple linear regression analysis. Similar results in respect of year and gender were found for students in a further 12 medical schools, although these results have to be interpreted with caution due to low response rates.

The results lead us to conclude that on the measures used in this study, in current UK medical education, there is no evidence that final year students are less empathetic than those starting the course. The results for New Zealand also demonstrate no significant differences in empathy between students starting and approaching the end of undergraduate medical education.

In line with general population studies, female students recorded higher mean scores for empathy, compared to male students [16]. Similarly, graduate entry course students, at a given point in their course, recorded higher mean scores than standard course students. Since they start medical education having completed a first degree, graduate entry course students tend to be older and many have more relevant life experience than standard course students. Inconsistent results concerning the trajectory of empathy during medical education have been reported by studies both of students entering medical education typically at aged 18 [18, 30, 32, 52] and of those who do so having completed a first degree [20, 25, 27]. However in our study, students completing their course did not record lower empathy scores than those starting their course regardless of whether they had entered medical education as standard or graduate course students.

The cross-sectional design adopted in this study sought to test the findings of our previous longitudinal study which examined 4 cohorts of students (2007–2010) on an annual basis and which found no marked reduction in empathy during undergraduate medical education in one institution using only the IRI [26]. The current study is, to the best of our knowledge, the only multi-centre study investigating medical student empathy in the UK with data generated from institutions using a variety of approaches to medical education within the overarching regulatory framework of the General Medical Council [53]. We also believe this to be the only study incorporating both UK and New Zealand data.

Analysis of the extent to which the type of course influences the development of empathy is not possible without detailed stratification of schools by course content and structure. Simple descriptions such as “problem based learning” and “integrated” may misrepresent the true nature of the course. Such stratification in a meaningful way was not practicable. As a result, we did not analyse our findings by ‘course type’.

A limitation of the study is the use of self-report scales, a weakness shared by similar studies. The extent to which scales actually measure empathy in clinical practice may be questioned, but there is evidence to support the validity of the scales used here; in addition, such measures are the only means of comparing large numbers of students [17, 39, 40, 44, 54]. Another weakness is the moderate response rate. It could be the case that students who were interested in the study and thus more likely to respond were more likely to be empathetic. A further weakness was the tendency for rates among final year students to be lower than those amongst first year students.

Studies of medical student empathy have produced mixed results. Some have found a reduction during undergraduate education [18, 23]. Other studies have found no change, or an increase [24, 26–32]. Results of studies investigating empathy in the wider health professional population are similarly mixed [35–38]. One danger of this confusion is a focus on short term initiatives aimed at enhancing empathy. There is some, limited, evidence that such interventions work in the short-term [55] but it can be suggested that there is a need to identify more precisely the factors that enhance or undermine empathy over longer periods.

Whilst US evidence suggests that empathy declines during internship [56, 57], similar evidence for the UK and elsewhere is scarce. A recent systematic review of communication skills in the postgraduate years highlighted the lack of consensus concerning their trajectory [58]. With greater potential for significant patient safety issues as they begin clinical practice, it would seem crucial to understand the trajectory of empathy among newly qualified doctors. Such understanding may help to inform choices facing those responsible for undergraduate medical education in order best to equip graduates for future practice.

## Conclusion

Participant male and female medical students approaching the end of their undergraduate education, whether on standard or graduate entry courses, did not record lower levels of empathy, compared to those at the beginning of their course. This finding of, at least, no reduction in empathy during medical studies before qualification is encouraging. Nevertheless, questions remain concerning the trajectory of empathy after qualification and how best to support it through the pressures of starting out in medical practice.

## Appendix 1

### Details of ethical approval

University of Cambridge: Psychology Research Ethics Committee application number: 2012.44. This approval was accepted under reciprocal arrangements by the following:

University of Birmingham,

University of Bristol,

University of Glasgow,

Hull Yorks Medical School,

University of Plymouth

Details of the ethical approval granted in other participating institutions:

Brighton and Sussex Medical School, University of Sussex: BSMS Research Governance and Ethics Committee (RGEG) application number: 13/163/MCD.

Cardiff University School of Medicine Research Ethics Committee application number: 12/57.

University of Exeter Medical School Ethics Committee application number: 13/009/033.

University of Galway, Ireland, Research Ethics Committee application number: 13/MAR/15.

King’s College, University of London, Biomedical Sciences, Dentistry, Medicine and Natural & Mathematical Sciences Research Ethics Subcommittee application number: BDM 13/14-14.

University of Leicester Medicine and Biological Sciences College Ethics Committee application number: jmh9-678b.

University of Liverpool School of Medicine Education Research Ethics Committee application number: 201306139.

University of Manchester Research Ethics Committees application number:14127.

University of Nottingham Faculty of Medicine and Health Sciences, Research Ethics Committee application number: G10102013 SoM PC.

University of Otago, Wellington, New Zealand: The Human Ethics Committee application number: D13/306.

University of Swansea College of Human and Health Sciences and College of Medicine Research Ethics Committee application number:11113.

## Appendix 2

### Comparison of demographic characteristics of students in the HRRG and LRRG schools

**Table 8:** Demographic characteristics of LRRG and HRRG schools’ respondents

	LRRG Schools		HRRG Schools	
	First	Final	First/second	Final
	Year	Year	Year	Year
All respondents	721	467	652	487
Female students	439 (60.9 %)	298 (63.8 %)	399 (61.2 %)	296 (60.7 %)
Graduate entry students	58 (8.0 %)	26 (5.6 %)	101 (15.5 %)	82 (16.8 %)
Non UK medical school students	76 (10.5 %)	34 (7.3 %)	165 (25.3 %)	140 (28.7 %)
Average Age (Years)				
Standard entry UK	19.1	24.1	18.7	25.1
Graduate entry UK	26.1	27.7	23.8	28.1
Non UK medical school students	19.2	24.7	20.5	23.4
Socioeconomic group				
HMO 1.1 & 1.2^a^	570 (79.2 %)	377 (80.7 %)	521 (80.2 %)	373 (76.6 %)

^a^UK National Statistics Socio-economic Classification: HMO: Higher Managerial Occupations eg company directors, doctors, teachers

### Comparison of mean empathy scores recorded by of students in the HRRG and LRRG schools

We used student t tests to compare the mean empathy scores recorded by students in the HRRG and LRRG schools. First year male students in the LRRG schools recorded slightly higher mean scores for IRI-EC than HRRG first/second year male students (t = 1.977, *p* = 0.049 Cohen’s d 0.171). Female final year students in the LRRG schools recorded slightly higher mean scores for IRI-PT than HRRG final/fifth year female students (*t* = 1.993, *p* = 0.047 Cohen’s d 0.164)
